# Inhibition of histone acetylation by curcumin reduces alcohol-induced fetal cardiac apoptosis

**DOI:** 10.1186/s12929-016-0310-z

**Published:** 2017-01-05

**Authors:** Xiaochen Yan, Bo Pan, Tiewei Lv, Lingjuan Liu, Jing Zhu, Wen Shen, Xupei Huang, Jie Tian

**Affiliations:** 1 Department of Cardiology, Heart Centre, The Children’s Hospital of Chongqing Medical University, 136 Zhongshan Er Rold, Yu Zhong District, Chongqing, 400014 China; 2 Children’s Hospital of Chongqing Medical University, Ministry of Education Key Laboratory of Child Development and Disorders, Chongqing, China; 3 China International Science and Technology Cooperation base of Child Development and Critical Disorders, Chongqing, China; 4 Chongqing Key Laboratory of Pediatrics, Chongqing, China; 5 Department of Biomedical Science, Charlie E. Schmidt College of Medicine, Florida Atlantic University, 777 Glades Road, Boca Raton, FL 33431 USA

**Keywords:** Alcohol, Apoptosis, Histone acetylation, Fetal cardiac development, Caspase

## Abstract

**Background:**

Prenatal alcohol exposure may cause cardiac development defects, however, the underlying mechanisms are not yet clear. In the present study we have investigated the roles of histone modification by curcumin on alcohol induced fetal cardiac abnormalities during the development.

**Methods and results:**

Q-PCR and Western blot results showed that alcohol exposure increased gene and active forms of caspase-3 and caspase-8, while decreased gene and protein of bcl-2. ChIP assay results showed that, alcohol exposure increased the acetylation of histone H3K9 near the promoter region of caspase-3 and caspase-8, and decreased the acetylation of histone H3K9 near the promoter region of bcl-2. TUNEL assay data revealed that alcohol exposure increased the apoptosis levels in the embryonic hearts. In vitro experiments demonstrated that curcumin treatment could reverse the up-regulation of active forms of caspase-3 and caspase-8, and down-regulation of bcl-2 induced by alcohol treatment. In addition, curcumin also corrected the high level of histone H3K9 acetylation induced by alcohol. Moreover, the high apoptosis level induced by alcohol was reversed after curcumin treatment in cardiac cells.

**Conclusions:**

These findings indicate that histone modification may play an important role in mediating alcohol induced fetal cardiac apoptosis, possibly through the up-regulation of H3K9 acetylation near the promoter regions of apoptotic genes. Curcumin treatment may correct alcohol-mediated fetal cardiac apoptosis, suggesting that curcumin may play a protective role against alcohol abuse caused cardiac damage during pregnancy.

## Background

During the past two decades, about 1.35 million children were born annually having congenital heart disease (CHD). The incidence of CHD is very high (9.3%) and accounts to one third of the total number of birth defects [1]. Thus, it has been a major concern in relation to public health. The causes of CHD are complex, only about 15% of CHD are known to stem from clear genetic reasons, while vast majority are caused by the interaction of environmental and genetic factors. One of the common environmental teratogenic factors is maternal alcohol abuse during pregnancy. Epidemiological data have shown that mothers consume alcohol during pregnancy and the rate of their offspring suffering from CHD would be doubled [2]. Animal studies have also shown that alcohol exposure can lead to congenital cardiac defects such as ventricular septal defects [3–5] and several types of cardiomyopathy [6]. Both in vivo and in vitro studies have shown that alcohol exposure can induce apoptosis of cardiomyocytes [6, 7].

Epigenetics modification plays a key role in the regulation of embryonic development and cardiogenesis [8–12]. Acetylation of histone generated by histone acetyltransferases (HATs) can neutralize the positive charges between histone and DNA allowing the binding of transcription factors to DNA, and consequently advances gene transcription [13, 14]. In addition, studies have also revealed that alcohol exposure can lead to hyperacetylation of histone H3K9 [15–17], an event believed to be associated with abnormal heart development [14, 18–21]. H3K9 plays a critical role in cell cycle progression and apoptosis [22, 23]. Pregnant mice expose to alcohol results in elevated levels of acetylated histone H3K9 followed by apoptosis in fetal lung [24], suggesting that increased acetylation of H3K9 could alter the expression of genes that induce apoptosis. Caspase-3 is a caspase protein that encoded by the caspase-3 gene, the protein itself can be cleaved and activated during apoptosis. And cleaved caspase-3 plays a central role in the execution-phase of cell apoptosis. Acetylation of histone H3 also regulate the cleaved caspase-3 level. Exposure of N27 dopaminergic cells to paraquat induced histone H3 acetylation and cleaved caspase-3 upregulation, while inhibition of HAT activity by anacardic acid significantly reduced paraquat-induced caspase-3 proteolytic cleavage [25].

Curcumin, the main constituent of the spice turmeric, is reported as the first naturel HAT inhibitor [26]. Several investigators have provided evidences suggesting that curcumin has protective effects against abnormal heart development [27]. In this study, we have measured the mRNA of caspase-3, caspase-8 and bcl-2, and the protein of cleaved caspase-3, cleaved caspase-8 and bcl-2. The purpose of the study is to understand whether histone H3K9 acetylation can regulate the cardiomyocytes apoptosis induced by alcohol. And, whether inhibition of histone H3K9 acetylation by curcumin, can reverse the over-expressed apoptosis genes.

## Methods

### Prenatal ethanol exposure

Fifty healthy adult C57BL6 mice (weight 21–28 g, specific pathogen free (SPF) grade) were purchased from the Experimental Animal Center of Chongqing Medical University (Chongqing, China). All experimentation procedures were conducted in accordance with the ethical guidelines provided by Chongqing Medical University on laboratory animals. Mice were raised in a stable environment (22 ± 1 °C, 55 ± 5% humidity) with 12 h’ light: 12 h’ dark cycle (light 19:00–07:00) and provided food ad libitum. After mating in the evening (5 pm), female mice were examined for the vaginal plug at 8:00 AM in the following morning. The day on which the vaginal plug was detected was considered as embryonic day 0.5 (E0.5). Between the embryonic day 7.5 (E7.5) and embryonic day 15.5 (E15.5), every morning at 8 am, the study mice were given ethanol (56% v/v in saline) using oral gavage at dosage of 10 μl/g while the controls were given isovolumetric, isocaloric glucose saline solution. The mice were monitored after treatment and it was seen that the study mice immediately appeared intoxicated and lethargic following ethanol exposure; however, they began moving around normally 35 mins after the exposure. At E 17.5, all pregnant mice were euthanized using carbon dioxide asphyxia and the embryonic hearts were promptly retrieved from the mouse pups.

### Culture and treatment of cardiac progenitor cells

The cardiac progenitor cells were purchased from Molecular Oncology Laboratory at the University of Chicago Medical Center [28]. The cells were cultured in Dulbecco’s modified Eagle medium (DMEM)/high glucose (Thermo, USA) supplemented with 10% fetal bovine serum (FBS) (Hyclone, USA), 100U/ml penicillin and 100 μg/ml streptomycin (Thermo, Waltham, MA, USA) in 37 °C humid air with 5% CO2. The cells were divided into five groups: alcohol; curcumin; alcohol + curcumin; DMSO; and control. From our previous studies [14, 18], we found that 200 mM alcohol was an effective concentration resulting in hyperacetylation of cardiac progenitor cells, and 25 μM curcumin could completely reversed the hyperacetylation induced by alcohol (200 mM). So we used 200 mM alcohol and 25 μM curcumin (SIGMA, USA) to intervene our cells for 24 h.

### Total RNA extraction and quantitative real-time PCR

Total RNA was extracted via RNA extraction kit (Bioteck, Beijing, China), then reverse transcribed into cDNA with oligo dT-adaptor primer and AMV reverse transcriptase kit (TaKaRa, Ostu, Japan). Quantitative real-time polymerase chain reaction (RT-PCR) was performed using SYBR Green RealMasterMix kit (Tiangen, Beijing, China) with the following parameters: pre-denaturation at 95 °C for 3 min, followed by 40 cycles of denaturation at 95 °C for 10s, annealing at proper temperature for 30s, elongation at 72 °C for 20s. The primers used in the study are listed in Table 1. The annealing temperatures were 56 °C for caspase-3, caspase-8, bcl-2 and β-actin. The relative mRNA levels for each gene were normalized to β-actin using the 2^-ΔΔCt^ (Ct is cycle threshold) method [29].

**Table 1:** The primers used in Q-PCR or CHIP assays

Q-PCR Primer			Produce size
Caspase-3	Forward	5'-TGACTGGAAAGCCGAAACTC-3'	101 bp
	Reverse	5'-GCAAGCCATCTCCTCATCA-3'	
Caspase-8	Forward	5'-GCCCTCAAGTTCCTGTGCT-3'	115 bp
	Reverse	5'-GATTGCCTTCCTCCAACATC-3'	
Bcl-2	Forward	5'-CGACTTCTTCAGCATCAGGA-3'	130 bp
	Reverse	5'-TGAGCCACAGGGAGGTTCT-3'	
β-actin	Forward	5'-GGAGATTACTGCCCTGGCTCCTA-3'	174 bp
	Reverse	5'-GACTCATCGTACTCCTGCTTGCTG-3'	
CHIP Primer			
Caspase-3	Forward	5'-AGTCCCTTACATCCAACG-3'	113 bp
	Reverse	5'-AGCACAGCGATCAGCATC-3'	
Caspase-8	Forward	5'-CAGAGGGATCAGGTTGGG-3'	102 bp
	Reverse	5'-AGGAGTCATAGCAAGGGT-3'	
Bcl-2	Forward	5'-TGTGGTTGCTGACACTTGAAC-3'	83 bp
	Reverse	5'-ACTCGTGGTTGGTGAGATGG-3'	

### Western blot

Western blotting assays were performed as previously reported [19, 21, 30]. The primary and secondary antibodies used are listed in Table 2. Specific target protein bands were revealed with enhanced chemiluminescence (Millipore, Billerica, USA), and analyzed using Quantity One Version 4.62 software (Bio-Rad, Richmond, CA). The β-actin was used as the internal control.

**Table 2:** The antibodies used in Western blotting assays

Primary antibody		Dilution	Secondary antibody (Boster 1:10000)
H3acK9	Abcam	1:500	anti-rabbit
Caspase-3	CST	1:1000	anti-rabbit
Cleaved caspase-3	Arigo	1:500	anti-mouse
Caspase-8	CST	1:1000	anti-rabbit
Cleaved caspase-8	CST	1:1000	anti-rabbit
Bcl-2	Santa Cruz Biotechnology	1:500	anti-mouse
β-actin	Abcam	1:2000	anti-mouse

### Chromatin immunoprecipitation (ChIP) assay

CHIP assays were performed as previously reported [21, 30, 31]. The primers used in the study are listed in Table 1. The annealing temperature was 56 °C for caspase-3, caspase-8 and bcl-2.

### TUNEL staining

TUNEL (Terminal-deoxynucleoitidyl Transferase Mediated Nick End Labeling) assay (KeyGEN, Nanjing, China) was used to assess the mode of apoptosis of alcohol treatment. Fifteen μm thick frozen section was fixed in 10% formalin for 20 min, then incubated in permeabilizing solution (0.1% Triton X in 0.1% phosphate-buffered saline) for 5 min at room temperature. After blocking in 0.3% H2O2 for 30 min, the frozen section was incubated with TdT (Terminal Deoxynucleotidyl Transferase) enzyme reaction mixture for 60 min at 37 °C, and then conjugated with streptavidin-HRP for 30 min at 37 °C. This was followed by incubation with 3’-diaminobenzidine (DAB) solution. Then counterstained the section with hematoxylin and the positively stained cells were photographed on a Nikon 800 photomicroscope.

### Apoptosis assay

Cardiac progenitor cells (1 × 10^6^/well), which had been treated with alcohol, curcumin, alcohol + curcumin or DMSO, were incubated with Annexin-V for 30 min followed by propidium iodide for 10 min. Subsequently, the apoptosis was subject to analysis using flow cytometer (BD FACSCalibur, USA).

### Statistical analysis

Statistical analysis was performed using mean ± SD or one-way analysis of variance (ANOVA). Value of *P* < 0.05 was considered to be statistically significant.

## Results

### Effect of alcohol exposure on pregnant mice

Pregnant C57 mice were exposed to alcohol (56% v/v in saline) in a dosage of 10 μl/g by gavage between E7.5 and E15.5. The peak blood-alcohol levels was 212.43 ± 56.53 mg/100 ml 40 min after alcohol exposure.

### Effect of alcohol exposure on H3K9 acetylation of embryonic hearts in E17.5

**Fig. 1:**
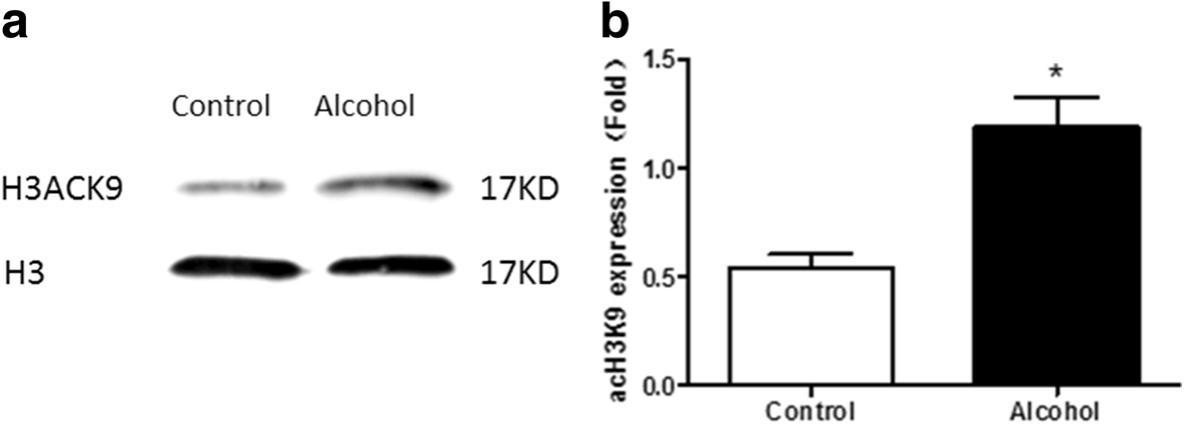
Effect of Alcohol Exposure on H3K9 Acetylation of Fetal Hearts in E17.5. **a** Western blot bands. **b** Prenatal alcohol exposure increased the acetylation of histone H3K9. *p < 0.05

In our previous study, we has discovered that alcohol exposure increased the levels of H3K9 acetylation in embryonic hearts between E11.5 and E18.5, and it reached its highest point in E17.5 [19]. In this study, using Western blotting assays, we found that H3K9 acetylation was increased significantly in embryonic hearts at E17.5 after alcohol exposure (Fig. 1).

### Effect of alcohol exposure on the expression of apoptosis gene of embryonic hearts in E17.5

**Fig. 2:**
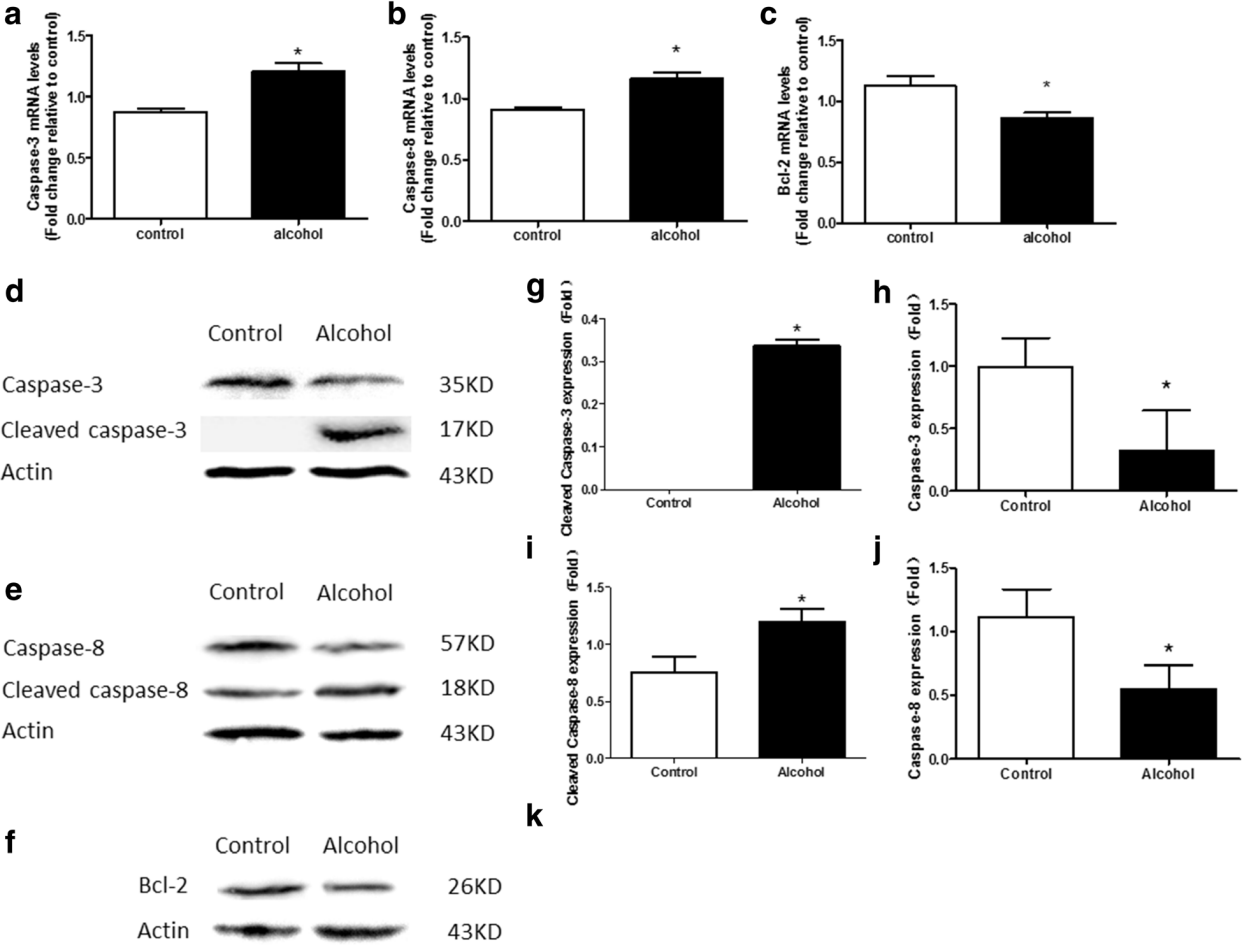
Effect of Alcohol Exposure on the Expression of Apoptosis Gene of Embryonic Hearts in E17.5. **a**, **b** The mRNA levels of caspase-3 and caspase-8 increased more with alcohol exposure, compared with the control group. **c** The mRNA levels of bcl-2 decreased more in the alcohol group than in the control group. **d**, **e**, **f** Western blot band. **g**, **h** Western blotting showed that prenatal alcohol exposure increased the expression of cleaved caspase-3 and decreased the expression of caspase-3. **i**, **j** Western blotting showed that pregnant alcohol exposure increased the expression of cleaved caspase-8 and decreased the expression of caspase-8. **k** Western blotting showed that bcl-2 significantly decreased in alcohol group than in control group. *p < 0.05

In order to detect the apoptosis inducted by alcohol treatment, we monitored caspase-3, caspase-8, and bcl-2 using Quantitative Real-Time PCR assays and Western blotting assays. Q-PCR data showed that mRNA of caspase-3 and caspase-8 in alcohol group was higher than that in control group (*P* < 0.05) (Fig. 2a, b). Whereas bcl-2 was decreased in the alcohol group compared to that in the control group (*P* < 0.05) (Fig. 2c). Furthermore, we examined cleaved and pro caspase-3, caspase-8 and bcl-2 using Western blotting assays. Cleaved caspase are small fragments extracted from the caspase, which play a key role in caspase-dependent pathway for apoptosis. The expression of caspase-3 (Fig. 2d, h), caspase-8 (Fig. 2e, j) and bcl-2 (Fig. 2f, k) was decreased in the alcohol group compared to that in the control group (*P* < 0.05). Whereas the cleaved caspase-8 was increased in alcohol group compared to that in the control group (*P* < 0.05) (Fig. 2e, i). However, the cleaved caspase-3 was only observed in alcohol group (Fig. 2d, g).

### Effect of alcohol exposure on myocardial apoptosis

**Fig. 3:**
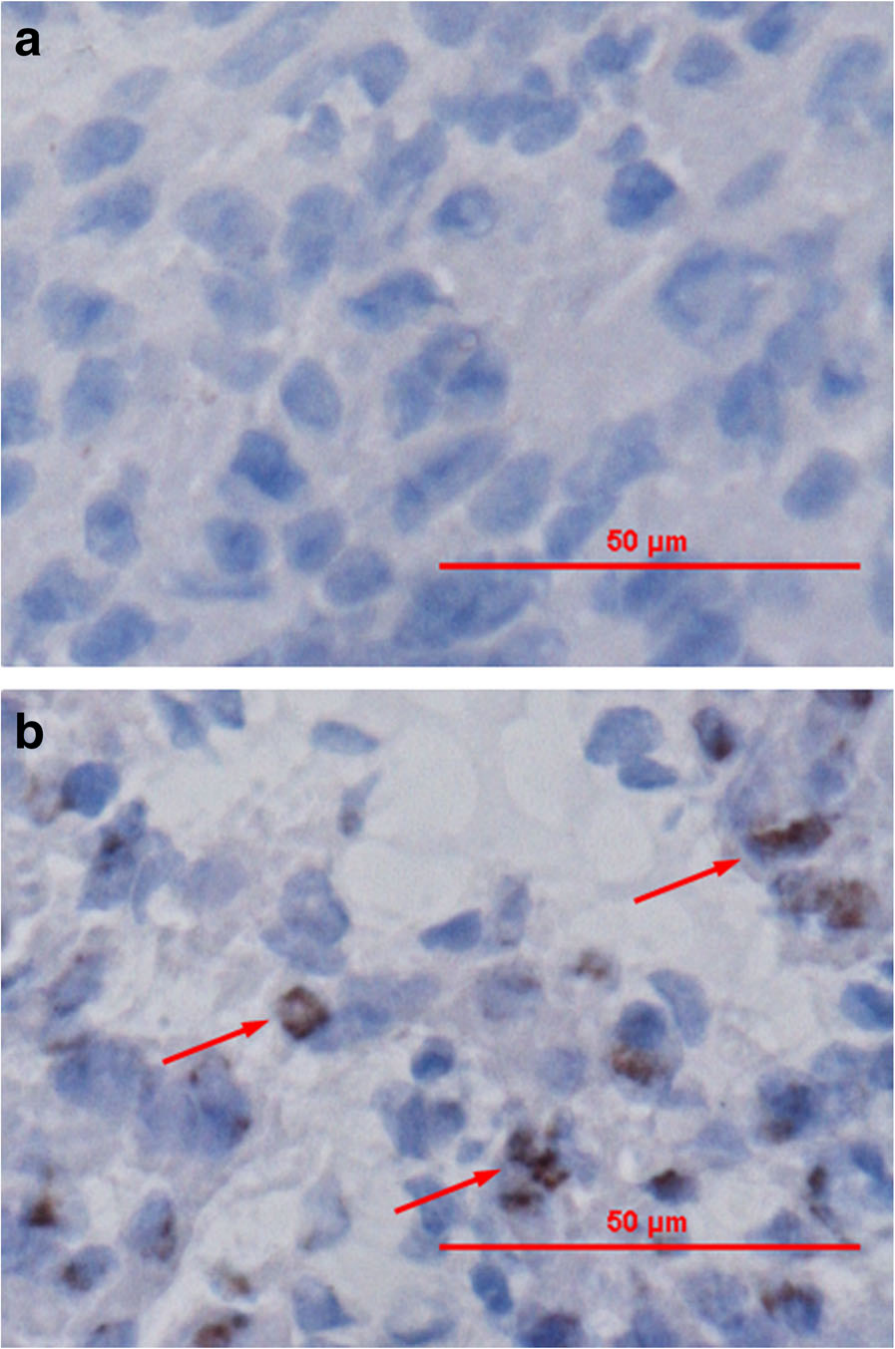
Effect of Alcohol Exposure on Myocardial Apoptosis. TUNEL assay results showed that alcohol exposure increased the apoptosis cells in the fetal heart. **a** Control group. **b** Alcohol group. TUNEL-positive cells are shown in brown (*arrow*)

TUNEL assay was used to assess the mode of apoptosis with alcohol treatment. As shown in Fig. 3, the positively stained cells in the alcohol group were significantly higher than those in the control group.

### Effect of alcohol exposure on acetylation of histone H3K9 near the promoter regions of apoptosis genes

**Fig. 4:**
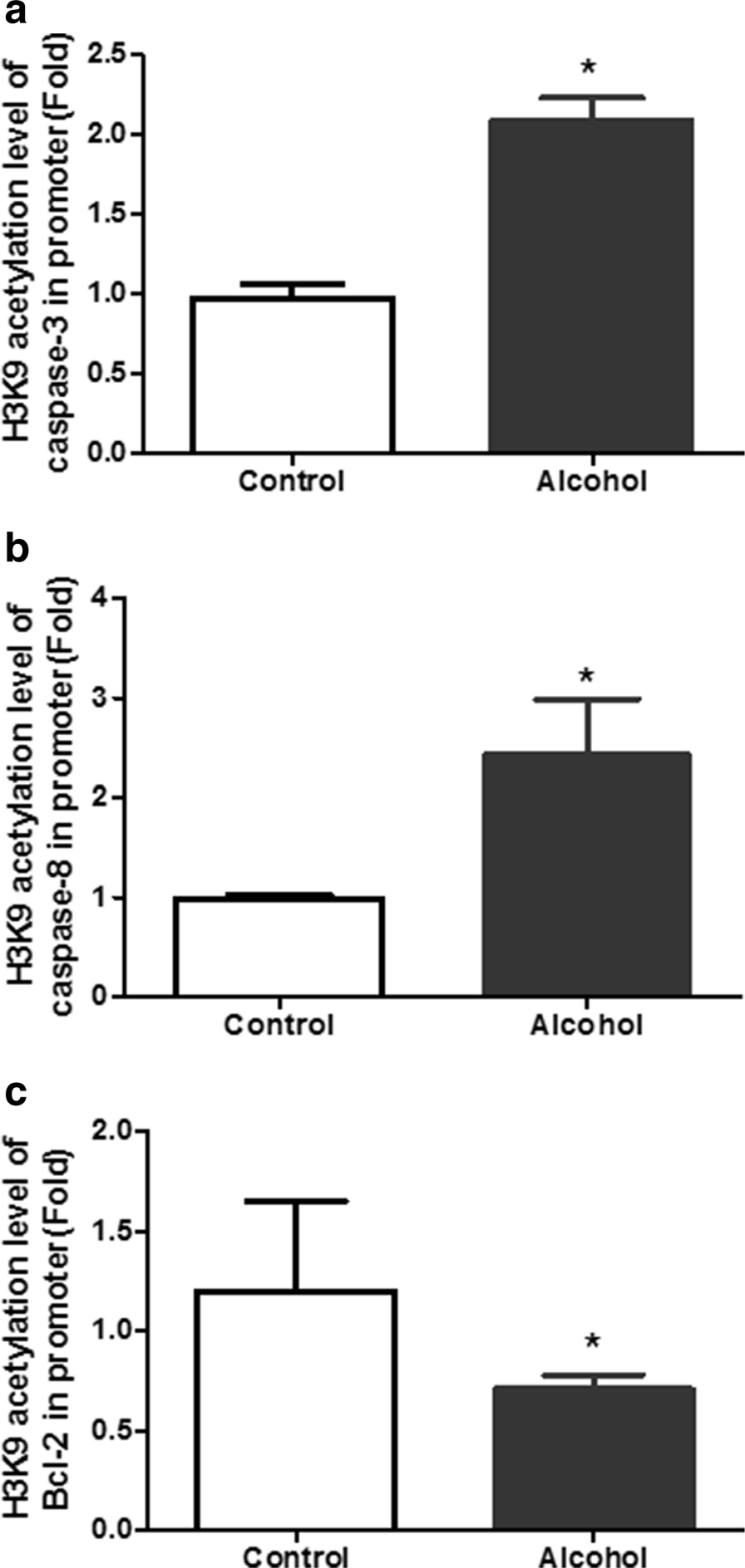
Effect of Alcohol Exposure on Acetylation of Histone H3K9 near the Promoter Regions of Apoptosis Genes. **a**, **b** ChIP-Q-PCR data showed that alcohol increased the acetylation of histone H3K9 near the promoter regions of caspase-3 and caspase-8. **c** ChIP-Q-PCR data showed that alcohol decreased the acetylation of histone H3K9 near the promoter region of bcl-2. *p < 0.05

In order to determine whether the change of caspase-3, caspase-8 and bcl-2 expressions following alcohol treatment were associated with hyperacetylation of histone H3K9 near the promoter regions of these genes, we assessed the levels of the histone H3K9 acetylation near the promoter regions of caspase-3, caspase-8 and bcl-2 using CHIP assays. We observed that alcohol could increase the acetylation level of histone H3K9 near the promoter regions of caspase-3 and caspase-8 (*p* < 0.05) (Fig. 4a, b). Meanwhile, alcohol significantly decreased the acetylation level of histone H3K9 near the promoter region of bcl-2 (*p* < 0.05) (Fig. 4c).

### Effect of curcumin on acetylation of histone H3K9 in cardiac progenitor cells treated by alcohol

**Fig. 5:**
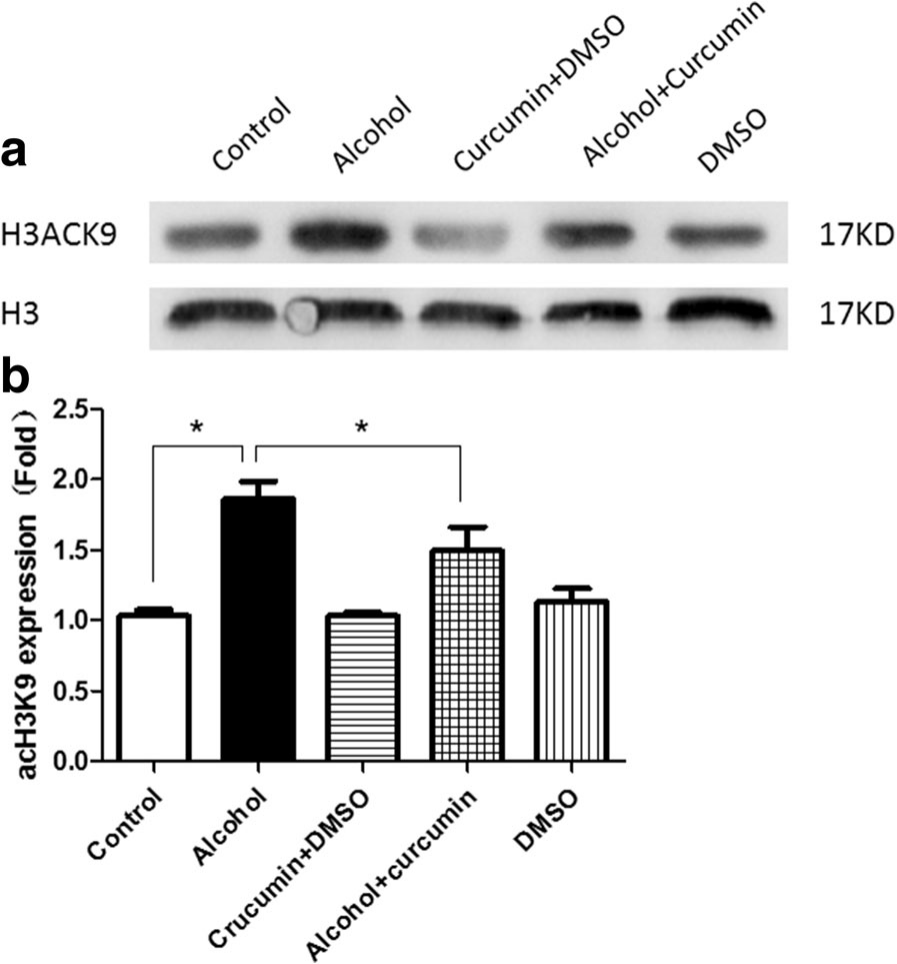
Effect of Curcumin on Acetylation of Histone H3K9 in Cardiac Progenitor Cells Treated by Alcohol. **a** Western blot bands. **b** Alcohol treatment lead to the hyper-acetylation of histone H3K9 in cardiac progenitor cells, and curcumin pretreatment could prevent this change. *p < 0.05

We analyzed whether the hyperacetylation of H3K9 could be reversed by curcumin. Alcohol increased acetylation of histone H3K9 but when treated with curcumin and alcohol simultaneously, the hyperacetylation was reversed (Fig. 5). However, the expression of acetylation of histone H3K9 had no effect when treated only with curcumin.

### Effect of curcumin on expression levels of apoptosis genes in cardiac progenitor cells treated by alcohol

**Fig. 6:**
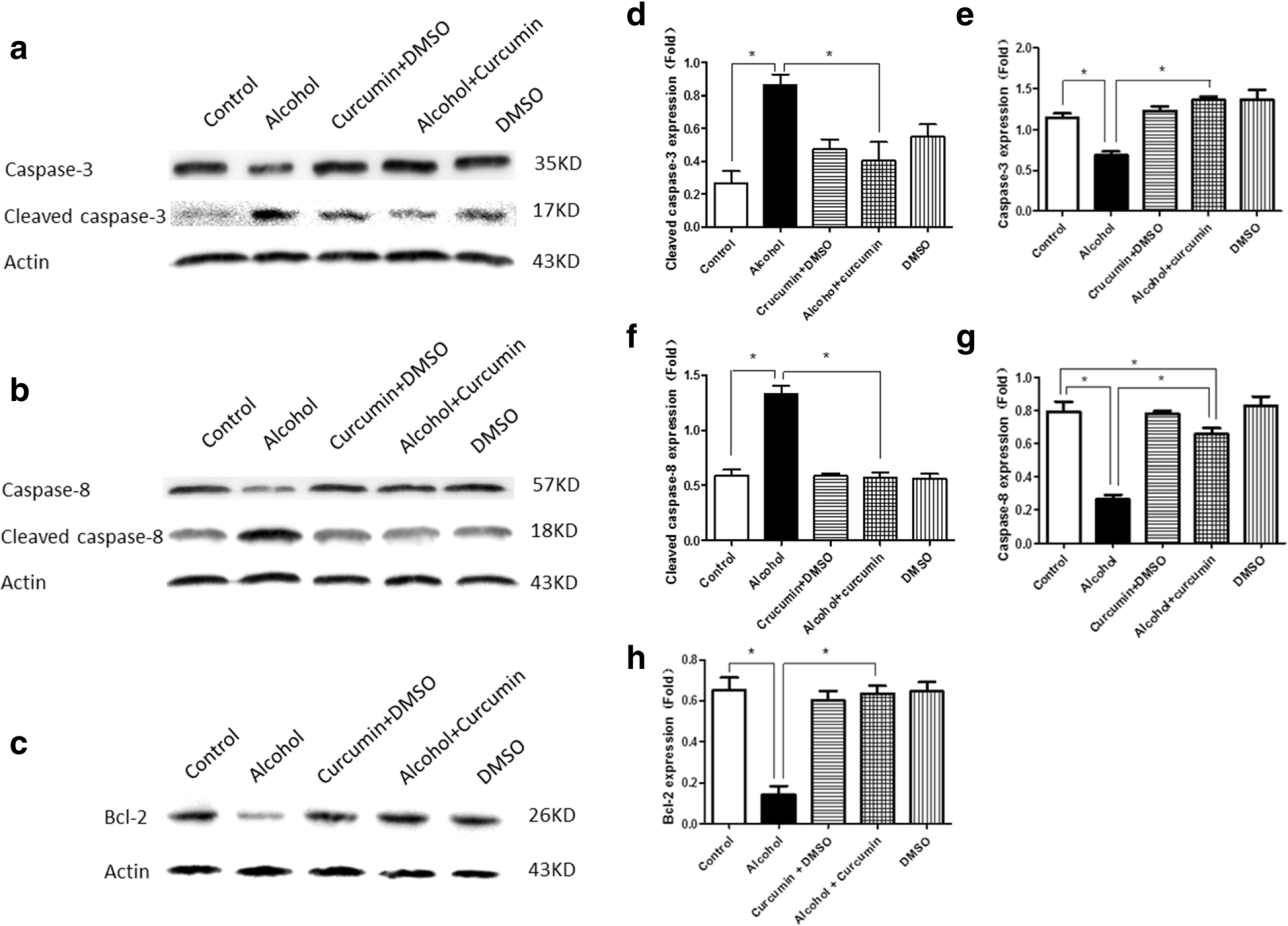
Effect of Curcumin on Expression Levels of Apoptosis Genes in Cardiac Progenitor Cells Treated with Alcohol. **a**, **b**, **c** Western blot band. **d**, **e** Western blotting showed that alcohol treatment increased the expression of cleaved caspase-3 and decreased the expression of caspase-3, and curcumin pretreatment could reverse this change. **f**, **g** Western blotting showed that alcohol treatment increased the expression of cleaved caspase-8 and decreased the expression of caspase-8, and curcumin pretreatment could reverse this change. **h** Alcohol treatment could decrease the expression of bcl-2 in cardiac progenitor cells, while curcumin pretreatment could prevent this change. *p < 0.05

We then analyzed whether the change of apoptosis genes could be reversed by curcumin. The results demonstrated that cleaved caspase-3 (Fig. 6a, d) and cleaved caspase-8 (Fig. 6b, f) levels increased significantly when treated with alcohol. At the same time, the expression of caspase-3 (Fig. 6a, e) and caspase-8 (Fig. 6b, g) along with the expression of bcl-2 (Fig. 6c, h) decreased. After the treatment with both curcumin and alcohol simultaneously, the over-expression of cleaved caspase-3 and cleaved caspse-8 were corrected. Moreover, the decrease in caspase-3, caspase-8 and bcl-2 were also reversed. However, curcumin did not affect the baseline level of cleaved caspase-3, cleaved caspase-8, caspase-3, caspase-8 and bcl-2.

### Effect of curcumin on apoptosis levels in cardiac progenitor cells treated by alcohol

**Fig. 7:**
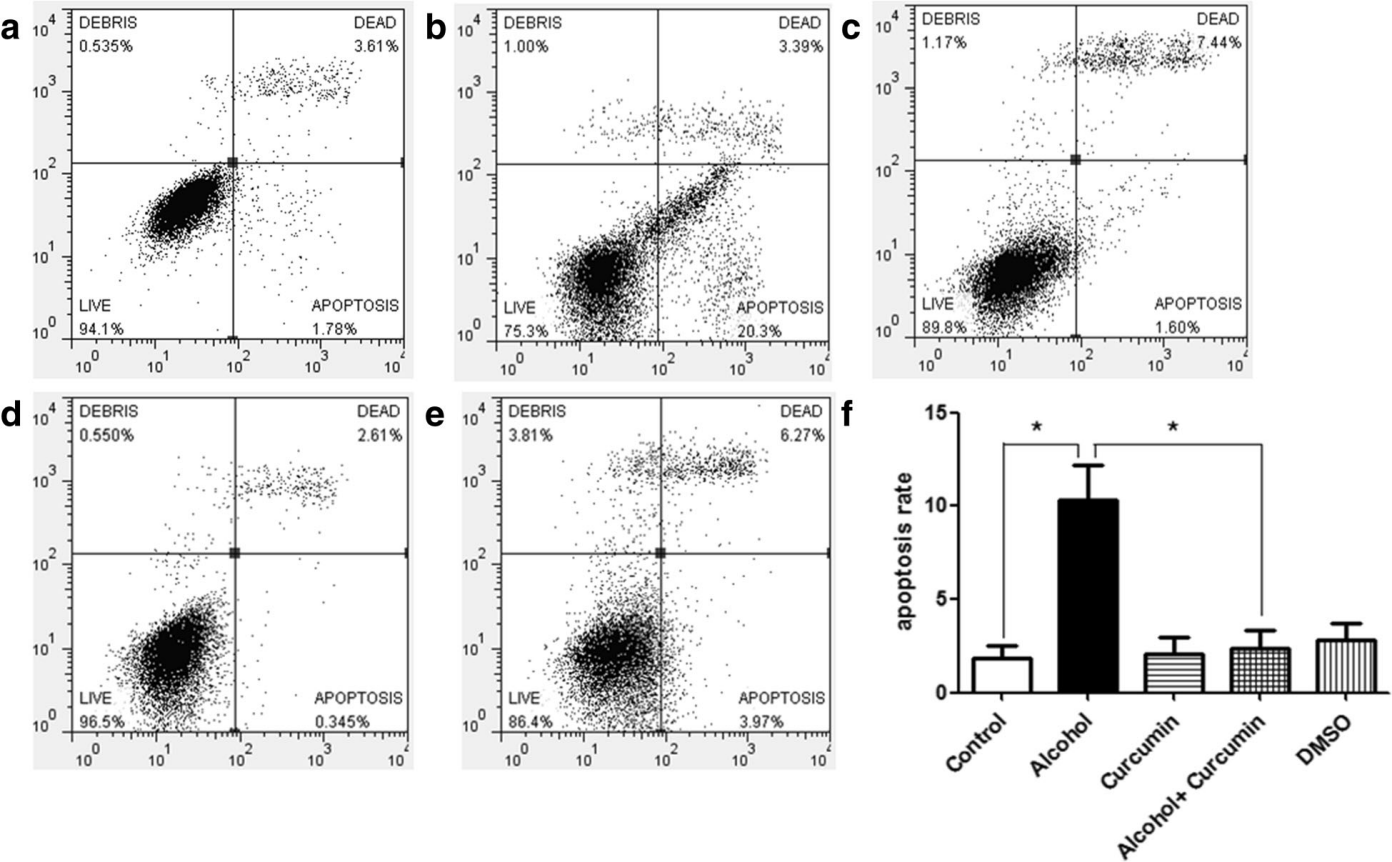
Effect of Curcumin on Apoptosis Levels in Cardiac Progenitor Cells Treated by Alcohol. Flow cytometry (FCM) assay was used to detect the apoptosis levels. **a** Control group. **b** Alcohol group. **c** Curcumin group. **d** Alcohol + Curcumin group. **e** DMSO group. **f** Quantitative analysis (percentage of apoptosis cells versus total cells) of five group showed that alcohol treatment increased the apoptosis rate and curcumin pretreatment could correct this change

Flow cytometry (FCM) assay was used to detect the apoptosis levels. As shown in Fig. 7, alcohol treatment increased the level of cardiac progenitor cells apoptosis rate to 10%, whereas curcumin intervention at an early stage could prevent this change.

## Discussion

Alcohol is a common environmental teratogenic factor and excessive use may generate excessive oxidative stress [32] and apoptosis in cardiomyocytes [6]. Prenatal alcohol exposure exerts a clear teratogenic effect on the developing heart, such as ventricular septal defect [3] and several types of cardiomyopathy [6]. Our previous study has also revealed that prenatal alcohol exposure could result in hypertrophy [21]. However, the underlying mechanisms are still unclear. It has been clearly established that cardiomyocytes are highly differentiated cells which rarely replicate after birth. Cardiomyocytes death (either necrosis or apoptosis) may be associated with abnormal heart development [33, 34]. Some studies have focused on the roles of apoptosis in heart development after prenatal alcohol exposure. Ren and co-workers [7] found that prenatal ethanol exposure increases apoptosis and alters myocardial contractile function in newborns. Goh et al. [35] found that, ethanol exposure during late gestation period may increase the expression of apoptosis genes, then accelerates the maturation of cardiomyocytes and increases cardiomyocyte and left ventricular (LV) tissue volume in the fetal heart. In this study, we find that prenatal alcohol exposure can increase the protein of cleaved caspase-3, cleaved caspase-8 and decrease the expression of bcl-2, consequently leading to cardiomyocytes apoptosis. Our data reveal that prenatal ethanol exposure promotes fetal myocyte apoptosis. This is an adverse event during heart development.

A body of evidence have shown that, histone acetylation is a key regulator during gene transcription [11, 36–38]. Histone acetylation regulates the expression of heart development related genes and plays a critical role in abnormal heart development when exposed to alcohol [30, 39, 40]. Although some researches show that HDACs can repress the growth of myocytes [41, 42], our previous study have demonstrated that alcohol exposure increased histone acetylation by enhancing HATs activities instead of altering HDACs [19]. Recent observations have further shown that cell death and survival are determined by the balance of histone acetylation/deacetylation [43–45]. For instance, the HDAC inhibitor induces histone hyperacetylation, which results in cancer cell death [46, 47]. On the contrary, anacardic acid, a HATs inhibitor, also has a neuroprotective effect against dieldrin-induced nigral dopaminergic neuronal degeneration [48]. So we detected the acetylation of histone H3K9 near the promoter regions of apoptosis genes. We find prenatal alcohol exposure can increase histone H3K9 acetylation near the promoter regions of caspase-3, caspase-8, while decrease histone H3K9 acetylation near the promoter region of bcl-2. And q-PCR data reveal that, alcohol can increase the mRNA of caspase-3, caspase-8 and decrease the mRNA of bcl-2. This data demonstrate that alcohol exposure can alter the balance of histone acetylation of apoptosis genes, which may cause the dysregulated expression of apoptosis genes. So, we emphasize that any alterations in the balance of histone acetylation in apoptosis genes may contribute to programmed cell death.

Curcumin is a curcuminoid found in turmeric, is reported as the first natural HATs inhibitor [26]. In our previous studies, we have confirmed that alcohol can impair the homeostasis of histone acetylation in cardiomyocytes, while this hyperacetylation can be reversed by curcumin. In this study, we found curcumin could down-regulate the hyper-acetylation of histone H3K9 induced by alcohol, but not back to the baseline level. This suggests that some factors such as the instability of curcumin and the turn-over rate of histone acetylation on those sites should be taken into account in the study. It is concluded that curcumin can indeed down-regulate partially the acetylation of histone H3K9 and correct the up-regulation of cleaved caspase-3, −8 as well as the down-regulation of bcl-2 induced by alcohol, which result in a correction of the high level of apoptosis in cardiac cells. These findings highlight the role of hyper-acetylation in the programmed cell death. We provide the implication that the increased histone acetylation could be seen as an early sign of cardiomyocytes apoptosis. To our knowledge, this is the first study of its kind that has investigated histone modification during the programmed cell death (apoptosis) in cardiomyocytes after alcohol exposure.

## Conclusions

Herein we giving some novel findings that alcohol induces hyperacetylation in histone H3K9 as an early event before cardiomyocytes apoptosis. It’s also unveiled that the HAT inhibitor curcumin had a protective effect against alcohol exposure, highlighting the translational potential of the drug while curing congenital heart disease. These results provide progression towards the understanding of alcohol abuse and epigenetic mechanisms in the pathogenesis of congenital heart disease. This surely will promote the development of new medicine for preventing congenital heart disease.

## Abbreviations

CHD: Congenital heart disease
ChIP: Chromatin immunoprecipitation
FCM: Flow cytometry
HAT: Histone acetyltransferase
HDAC: Histone deacetylase inhibitors
TUNEL: Terminal deoxynucleoitidyl Transferase Mediated Nick End Labeling

## References

[1] van der Linde D (2011). Birth prevalence of congenital heart disease worldwide: a systematic review and meta-analysis. J Am Coll Cardiol.

[2] Grewal J (2008). Maternal periconceptional smoking and alcohol consumption and risk for select congenital anomalies. Birth Defects Res A Clin Mol Teratol.

[3] Webster WS (1984). Alcohol and congenital heart defects: an experimental study in mice. Cardiovasc Res.

[4] Kvigne VL (2004). Characteristics of children who have full or incomplete fetal alcohol syndrome. J Pediatr.

[5] May PA (2007). The epidemiology of fetal alcohol syndrome and partial FAS in a South African community. Drug Alcohol Depend.

[6] Chen DB, Wang L, Wang PH (2000). Insulin-like growth factor I retards apoptotic signaling induced by ethanol in cardiomyocytes. Life Sci.

[7] Ren J (2002). Influence of prenatal alcohol exposure on myocardial contractile function in adult rat hearts: role of intracellular calcium and apoptosis. Alcohol Alcohol.

[8] Ohtani K, Dimmeler S (2011). Epigenetic regulation of cardiovascular differentiation. Cardiovasc Res.

[9] Connelly JJ (2013). Epigenetic regulation of COL15A1 in smooth muscle cell replicative aging and atherosclerosis. Hum Mol Genet.

[10] Hon GC (2013). Epigenetic memory at embryonic enhancers identified in DNA methylation maps from adult mouse tissues. Nat Genet.

[11] Yu CE (2013). Epigenetic signature and enhancer activity of the human APOE gene. Hum Mol Genet.

[12] Mathiyalagan P (2014). Interplay of chromatin modifications and non-coding RNAs in the heart. Epigenetics.

[13] Hasan S, Hottiger MO (2002). Histone acetyl transferases: a role in DNA repair and DNA replication. J Mol Med (Berl).

[14] Wang L (2012). Inhibition of histone acetylation by curcumin reduces alcohol-induced expression of heart development-related transcription factors in cardiac progenitor cells. Biochem Biophys Res Commun.

[15] Park PH, Miller R, Shukla SD (2003). Acetylation of histone H3 at lysine 9 by ethanol in rat hepatocytes. Biochem Biophys Res Commun.

[16] Kim JS, Shukla SD (2006). Acute in vivo effect of ethanol (binge drinking) on histone H3 modifications in rat tissues. Alcohol Alcohol.

[17] Choudhury M, Shukla SD (2008). Surrogate alcohols and their metabolites modify histone H3 acetylation: involvement of histone acetyl transferase and histone deacetylase. Alcohol Clin Exp Res.

[18] Zhong L (2010). Ethanol and its metabolites induce histone lysine 9 acetylation and an alteration of the expression of heart development-related genes in cardiac progenitor cells. Cardiovasc Toxicol.

[19] Pan B (2014). Alcohol consumption during gestation causes histone3 lysine9 hyperacetylation and an alternation of expression of heart development-related genes in mice. Alcohol Clin Exp Res.

[20] Peng C (2014). Inhibition of histone H3K9 acetylation by anacardic acid can correct the over-expression of Gata4 in the hearts of fetal mice exposed to alcohol during pregnancy. PLoS One.

[21] Peng C (2015). Alcohol-induced histone H3K9 hyperacetylation and cardiac hypertrophy are reversed by a histone acetylases inhibitor anacardic acid in developing murine hearts. Biochimie.

[22] Chen H, Tini M, Evans RM (2001). HATs on and beyond chromatin. Curr Opin Cell Biol.

[23] Li J (2002). Arsenic trioxide promotes histone H3 phosphoacetylation at the chromatin of CASPASE-10 in acute promyelocytic leukemia cells. J Biol Chem.

[24] Wang X (2010). Acute alcohol exposure induces apoptosis and increases histone H3K9/18 acetylation in the mid-gestation mouse lung. Reprod Sci.

[25] Song C (2011). Paraquat induces epigenetic changes by promoting histone acetylation in cell culture models of dopaminergic degeneration. Neurotoxicology.

[26] Devipriya B, Kumaradhas P (2013). Molecular flexibility and the electrostatic moments of curcumin and its derivatives in the active site of p300: a theoretical charge density study. Chem Biol Interact.

[27] Sun H (2014). Curcumin-mediated cardiac defects in mouse is associated with a reduced histone H3 acetylation and reduced expression of cardiac transcription factors. Cardiovasc Toxicol.

[28] Zhu GH (2009). Activation of RXR and RAR signaling promotes myogenic differentiation of myoblastic C2C12 cells. Differentiation.

[29] Livak KJ, Schmittgen TD (2001). Analysis of relative gene expression data using real-time quantitative PCR and the 2(−Delta Delta C(T)) Method. Methods.

[30] Gao W (2015). Alcohol exposure increases the expression of cardiac transcription factors through ERK1/2-mediated histone3 hyperacetylation in H9c2 cells. Biochem Biophys Res Commun.

[31] Sun H (2010). Inhibition of p300-HAT results in a reduced histone acetylation and down-regulation of gene expression in cardiac myocytes. Life Sci.

[32] Choudhury M (2010). Evidence for the role of oxidative stress in the acetylation of histone H3 by ethanol in rat hepatocytes. Alcohol.

[33] Hallaq H (2004). A null mutation of Hhex results in abnormal cardiac development, defective vasculogenesis and elevated Vegfa levels. Development.

[34] Wang X (2005). Targeted deletion of mek5 causes early embryonic death and defects in the extracellular signal-regulated kinase 5/myocyte enhancer factor 2 cell survival pathway. Mol Cell Biol.

[35] Goh JM (2011). Alcohol exposure during late gestation adversely affects myocardial development with implications for postnatal cardiac function. Am J Physiol Heart Circ Physiol.

[36] Yin W (2007). Histone acetylation at the human beta-globin locus changes with developmental age. Blood.

[37] Gupta A (2008). The mammalian ortholog of Drosophila MOF that acetylates histone H4 lysine 16 is essential for embryogenesis and oncogenesis. Mol Cell Biol.

[38] Chaturvedi P, Tyagi SC (2014). Epigenetic mechanisms underlying cardiac degeneration and regeneration. Int J Cardiol.

[39] Si L (2014). Smad4 mediated BMP2 signal is essential for the regulation of GATA4 and Nkx2.5 by affecting the histone H3 acetylation in H9c2 cells. Biochem Biophys Res Commun.

[40] Zhang W (2014). Prenatal alcohol exposure causes the over-expression of DHAND and EHAND by increasing histone H3K14 acetylation in C57 BL/6 mice. Toxicol Lett.

[41] McKinsey TA, Zhang CL, Olson EN (2002). Signaling chromatin to make muscle. Curr Opin Cell Biol.

[42] Zhang CL (2002). Class II histone deacetylases act as signal-responsive repressors of cardiac hypertrophy. Cell.

[43] Somech R, Izraeli S, J Simon A (2004). Histone deacetylase inhibitors--a new tool to treat cancer. Cancer Treat Rev.

[44] Marchion D, Munster P (2007). Development of histone deacetylase inhibitors for cancer treatment. Expert Rev Anticancer Ther.

[45] Soriano FX (2009). Role of histone acetylation in the activity-dependent regulation of sulfiredoxin and sestrin 2. Epigenetics.

[46] Aron JL (2003). Depsipeptide (FR901228) induces histone acetylation and inhibition of histone deacetylase in chronic lymphocytic leukemia cells concurrent with activation of caspase 8-mediated apoptosis and down-regulation of c-FLIP protein. Blood.

[47] Condorelli F (2008). Inhibitors of histone deacetylase (HDAC) restore the p53 pathway in neuroblastoma cells. Br J Pharmacol.

[48] Song C (2010). Environmental neurotoxic pesticide increases histone acetylation to promote apoptosis in dopaminergic neuronal cells: relevance to epigenetic mechanisms of neurodegeneration. Mol Pharmacol.

